# Stage-Stratified Analysis of Prognostic Significance of Tumor Size in Patients with Gastric Cancer

**DOI:** 10.1371/journal.pone.0054502

**Published:** 2013-01-30

**Authors:** Hongliang Zu, Feng Wang, Yan Ma, Yingwei Xue

**Affiliations:** Department of Gastroenterologic Surgery, The Third Affiliated Hospital of Harbin Medical University, Harbin, China; The University of Hong Kong, Hong Kong

## Abstract

**Background:**

The prognostic significance of tumor size in gastric cancer is not well defined. The objective of this study was to identify the prognostic value of tumor size in patients with gastric cancer.

**Methods:**

We retrospectively reviewed a total of 1800 patients with gastric cancer admitted to our hospital between 1997 and 2007. These patients were divided into two groups according to tumor size: small size group (SSG, tumor ≤5 cm) and large size group (LSG, tumor >5 cm). We compared clinico-pathologic features of the two groups and investigated the prognostic factors by performing univariate, multivariate, and stage- stratified analyses according to tumor size.

**Results:**

LSG had more aggressive clinico-pathologic features than SSG. Tumor size was an independent prognostic indicator in patients with gastric cancer. In a stratified-pT, pN, and pTNM analysis, survival of patients with LSG was significantly worse than that of patients with SSG and advanced stage. Tumor size was not a significant predictor of survival in patients with early stage tumors. Large tumor size was associated with shorter survival in patients with stages N0, N1, N2, and N3, and stages I, II, III, and IV.

**Conclusions:**

Tumor size is a simple and practical prognostic factor in patients with gastric cancer. Tumor size could supplement clinical staging in the future.

## Introduction

Although its incidence rate has steadily declined in recent decades, gastric cancer (GC) (stomach adenocarcinoma) remains a global health problem. Gastric carcinoma is the fourth most common malignancy in the world, with an estimated 989,000 new cases and 738,000 deaths reported in 2008. Over 70% of new cases and deaths occurred in developing countries, compared with an estimated 21,500 new cases and 10,880 new deaths in the Unites States in the same year [1], [2]. The identification of prognostic factors for gastric cancer is extremely important. Pre-operative or intra-operative prognostic factors could contribute to treatment planning. The depth of invasion and the presence of lymph node metastases are the most important prognostic factors in gastric cancer [3], [4], [5], [6]. This data is usually not available during surgery. Tumor size refers to the maximum diameter of the tumor that can be measured before or during surgery. It is used to predict a safe surgical margin and the required extent of extragastric resection. Although tumor size has an effect on the patient’s surgical management, the prognostic value of tumor size in patients with gastric cancer remains is not well defined. Some authors [7], [8] have demonstrated that tumor size was an independent prognostic indicator in gastric cancer, while other studies [9], [10] reported that tumor size was not an independent prognostic factor in patient survival. We evaluated the prognostic significance of tumor size in patients with gastric carcinoma. We performed a stratified-pT, pN and pTNM prognostic analysis to provide insight into the value of tumor size in patients with gastric cancer.

## Patients and Methods: A, B

### Patients and Methods: A

**Figure 1:**
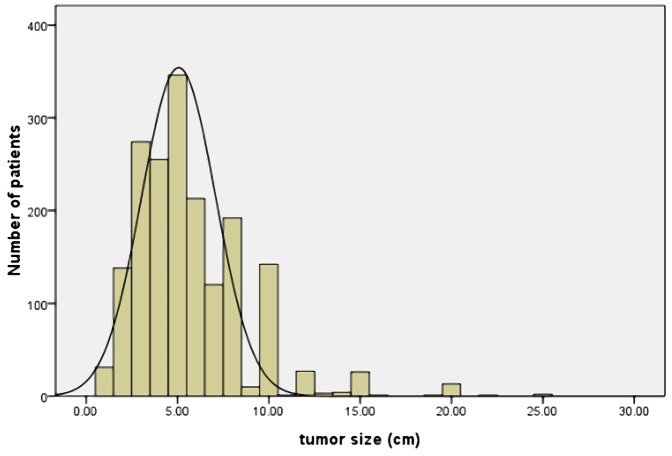
The distribution of number of patients ralated to tumor size. Tumor size ranged from 0.5 to 25 cm (mean 5.68 cm, median 5.0 cm).

Between 1997 and 2007, 1800 patients with histologically proven primary gastric adenocarcinoma underwent gastrectomy at the department of Surgical Gastroenterology, Affiliated Tumor Hospital of Harbin Medical University, Harbin, China. This retrospective study was approved by the *Ethics Committee* of Harbin Medical University and all patients signed an informed consent. All patients did not receive pre-operative chemotherapy or radiotherapy. Tumor size was measured according to the Japanese Classification of Gastric Cancer. The dissected stomach specimen was fixed on a flat board, and the maximum tumor diameter was determined. The distribution of tumor size is shown in Fig. 1. Tumors measured from 0.5 to 25 cm (mean: 5.68 cm). To determine an optimal tumor size threshold, survival rates were evaluated at 1-cm intervals. Survival rates were then compared with the established threshold using a log-rank test and and Cox stepwise proportional hazard test. The threshold value for tumor size was identified as the test size with the maximum X2 value (Table 1). The largest chi-square value was associated with a disease-specific survival of 278.1 ((P<0.001,) 95%CI =  (39.274–58.726) ) in the log-rank test and 60.3 ((P<0.001,) 95%CI =  (1.557–2.101)) in the Cox stepwise proportional hazard test, respectively. And determined the 5 cm in tumor size as optimal cut-off value. Based on this result, 1800 patients were divided into two groups: a small size group (SSG, tumor size ≤5 cm, n = 1,044) and a large size group (LSG, tumor size >5 cm, n = 756). The clinico-pathologic features and prognostic differences between patients with SSG and LSG were reviewed. The clinico-pathologic data were obtained from patient’s operative and pathological reports. Data included gender, age (≤60 years or >60 years), tumor location (upper, middle, lower, entire, X), gross appearance (Borrmann I, II, III, IV, X), surgical result (curative, non-curative), degree of differentiation (well differentiated, moderately differentiated, poorly differentiated, mucinous carcinoma, signet ring cell carcinoma: if there were two or more histological types, the histological type was defined by the predominant type), liver metastases (yes vs. no), combined organ resection (yes or no), total gastrectomy (yes or no), depth of tumor invasion (T1: tumor has invaded the mucosa or submucosa layer; T2: tumor has invaded the muscular layer or the subserosa; T3: tumor has invaded the serosa or penetrating serosa; T4: tumor invaded adjacent organs), 7th American Joint Committee on Cancer (AJCC) lymph node status (N0, N1, N2, N3a, N3b) and TNM stage (I, II, III, IV). Surgery was deemed curative when there was no gross residual tumor (including negative resection margins and no evidence of distant metastasis). Surgery was considered non-curative when tumor was present at any margin. Laparotomy and bypass procedures were excluded from the scope of this study.

**Table 1:** Chi-square value and P value by tumor size (log-rank test and Cox stepwise proportional hazard test).

Threshold	Log-rank test			Cox stepwise proportional hazard test		
	X^2^ value	P value	5-Year survival(>5 cm)	X^2^ value	P value	Hazard ratio (95% CI)
1 cm	11.501	0.001	46.1	1.876	0.171	3.953(0.553,28.248)
2 cm	74.232	0.000	43.3	11.619	0.001	2.353(1.439,3.849)
3 cm	150.651	0.000	34.0	22.108	0.000	1.748(1.385,2.206)
4 cm	189.385	0.000	34.0	30.915	0.000	1.627(1.370,1.932)
5 cm	278.115	0.000	26.1	60.279	0.000	1.809(1.557,2.101)
6 cm	220.752	0.000	24.8	26.724	0.000	1.530(1.311,1.785)
7 cm	195.257	0.000	22.6	29.106	0.000	1.499(1.389,1.618)
8 cm	157.061	0.000	17.1	23.477	0.000	1.558(1.302,1.865)
9 cm	148.531	0.000	17.0	20.174	0.000	1.521(1.267,1.826)
10 cm	61.214	0.000	13.0	11.115	0.001	1.609(1.217,2.128)
12 cm	87.593	0.000	6.0	20.652	0.000	2.183(1.559,3.056)
13 cm	71.714	0.000	6.0	20.49	0.000	2.224(1.574,3.144)
14 cm	82.623	0.000	3.9	22.692	0.000	2.376(1.664,3.392)

### Patients and Methods: B, Follow-up and Statistical Analysis

*Patient follow*-*up* lasted *until death or the cut*-*off date* of December 7, 2011. For patients who remained alive, data were censored at the date of the last contact. Only the patients who died of gastric cancer were regarded as tumor-related death cases. Chi-squared and Fisher exact tests were used for analyzing associations between categorical variables. Survival data were estimated using the Kaplan–Meier method. The log-rank test was used to compare differences in survival rates between different tumor size subgroups. Multivariate analyses of prognostic factors related to overall survival were carried out using the Cox stepwise proportional hazards test. A stratified univariate and multivariate analysis of tumor size, stage pT, pN and pTNM was performed to evaluate the impact of stage on prognosis. The criterion for statistical significance was p<0.05. All data analysis was performed using SPSS (SPSS Inc., Chicago, IL, USA) for Windows, Version 17.0.

## Results

### Result 1: Clinicopathologic Features

Among the 1800 patients identified, there were 1327 men (73.7%) and 473 women (26.3%). The mean age was 56.3 years (range: 24–80 years). Table 2 shows clinico-pathologic characteristics of 1044 (58%) patients in the SSG and 756 (42%) patients in the LSG. There were statistically significant differences in tumor location, gross appearance, histologic type, depth of invasion, presence of lymph node metastases, curative resection and TMN stage. LSG tumors were more frequently located in the entire stomach (18.5% vs. 1.9%), had a higher proportion of Borrmann type IV (12.4% vs. 4.0%) tumors, and had more mucinous carcinoma cell types (9.4% vs. 4.7%) than SSG tumors. SSG tumors were more often Borrmann type I or II, located in the distal stomach, and well differentiated. Tumor size was significantly related to depth of invasion and presence of lymph node metastases. LSG had a larger proportion of T3 (55.3% vs. 48.3%), T4 (32.4% vs. 8.3%), N3a (23.9% vs. 11.6%), and N3b (9.0% vs. 2.7%) tumors. SSG had a larger percent of T1 (12.6% vs. 0.8%), T2 (30.8% vs. 11.5%) and N0 (43.8% vs. 20.1%) tumors. Liver metastases were observed more frequently in LSG than in SSG patients (P<0.01). Patients with LSG tumors received more total gastrectomies (42.7% vs. 11.0%) and combined resections (9.0% vs. 2.3%) than patients with SSG. Gender and age were not significantly different between the two groups.

**Table 2:** Clinicopathological and treatment related factors between the LSG and SSG.

Variable	#SSG(n = 1044)	#LSG(n = 756)	P value
Gender			0.089
Male	754(72.2%)	573(75.8%)	
Female	290(27.8%)	183(24.2%)	
Age			0.148
Less than 60 years	632(60.5%)	432(57.1%)	
More than 60 years	412(39.5%)	324(42.9%)	
Degree ofdifferentiation			0.000
Well	38(3.6%)	15(2.0%)	
Moderate	400(38.3%)	256(33.9%)	
Poor	467(44.7%)	347(45.9%)	
Mucinous	49(4.7%)	71(9.4%)	
Signet	90(8.6%)	67(8.9%)	
Borrmann type			0.000
I	108(10.3%)	93(12.3%)	
II	167(16.0%)	36(4.8%)	
III	649(62.2%)	519(68.7%)	
IV	42(4.0%)	94(12.4%)	
Unknow	78(7.5%)	14(1.9%)	
Location			0.000
Upper	112(10.79%)	89(11.8%)	
Middle	135(12.9%)	176(23.3%)	
Lower	759(72.7%)	328(43.4%)	
Entire	20(1.9%)	140(18.5%)	
Unknow	18(1.7%)	23(3.0%)	
Stage T *			0.000
T1	132(12.6%)	6(0.8%)	
T2	321(30.8%)	87(11.5%)	
T3	504(48.3%)	418(55.3%)	
T4	87(8.3%)	245(32.4%)	
Stage N +			0.000
N0	457(43.8%)	152(20.1%)	
N1	231(22.1%)	154(20.4%)	
N2	207(19.8%)	201(26.6%)	
N3a	121(11.6%)	181(23.9%)	
N3b	28(2.7%)	68(9.0%)	
Stage TNM			0.000
I	294(28.2%)	24(3.2%)	
II	265(25.4%)	122(16.1%)	
III	416(39.8%)	415(54.9%)	
IV	69(6.6%)	195(25.8%)	
Curability			0.000
Yes	861(82.5%)	459(60.7%)	
No	183(17.5%)	297(39.3%)	
Liver metastasis			0.007
Yes	18(1.7%)	31(4.1%)	
No	1026(98.3%)	725(95.9%)	
Combinedresection			0.000
Yes	24(2.3%)	68(9.0%)	
No	1020(97.7%)	688(91.0%)	
Total gastrectomy			0.000
Yes	115(11.0%)	323(42.7%)	
No	929(89.0%)	433(57.3%)	

# SSG: small size group; LSG: large size group.

* T1: tumor has invaded the mucosa or submucosa layer; T2: tumor has invaded the muscular layer or the subserosa; T3: tumor has invaded the serosa or penetrating serosa; T4: tumor invaded adjacent organs.

+ N0: no regional lymph node metastasis; N1: 1–2 regional lymph node metastasis; N2: 3–6 regional lymph node metastasis; and N3a: 7–15 regional lymph node metastasis; N3b: ≥15 regional lymph node metastasis.

### Result 2: Univariate and Multivariate Survival Analysis

**Figure 2:**
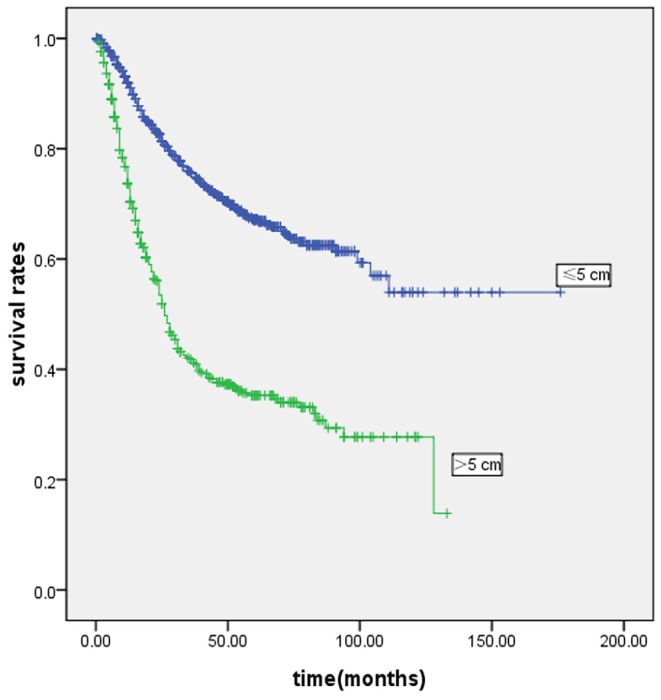
Kaplan-Meier survival curves in curative gastrectomy patients according to tumor size (n = 1320). Prognosis of larger tumor size was worse than smaller tumor size in patients with gastric cancer(P<0·001). Five-year survival rates were 44.0% and 70.7% in LSG and SSG, respectively.

**Figure 3:**
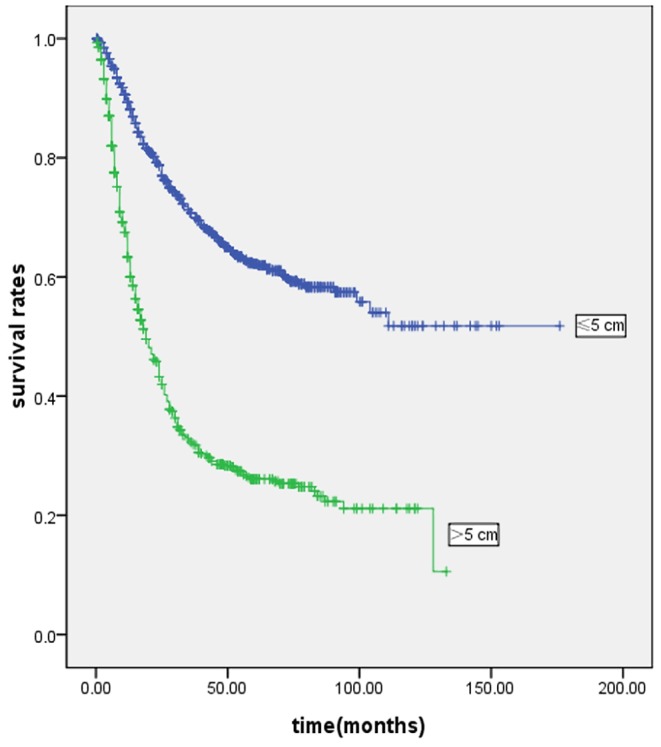
Kaplan-Meier survival curves in all underwent gastrectomy patients according to tumor size (n = 1800). Prognosis of larger tumor size was worse than smaller tumor size in patients with gastric cancer (P<0·001). Five-year survival rates were 26.1% and 62.1% in LSG and SSG, respectively.

The mean follow-up was 35 months (range: 1–176 months). Figures 2 and 3 show the survival curves of patients who underwent curative gastrectomy or gastrectomy. The 5-year survival rate was significantly lower in LSG patients than in SSG patients. Significant prognostic factors included age, gender, histological type, Borrmann type, tumor location, pT and pN stage, TNM stage, and curability. The Cox proportional hazards test revealed that tumor size, age, lymph node metastases and depth of invasion were independent prognostic factors in the patients who underwent curative gastrectomy (Table 3) or gastrectomy (Table 4).

**Table 3:** Multivariate Cox stepwise proportional hazard test for overall survival in 1320 gastric cancer patients with curative gastrectomy.

Variable	?2	P value	Hazard ratio (95% CI)
Age	22.593	<0·001	1.154 (1.023, 1.302)
Tumor size	34.574	<0·001	1.762 (1.459,2. 127)
Lymph node metastasis	114.335	<0·001	1.522 (1.409.1.644)
Depth of invasion	35.742	<0·001	1.565 (1.351,1.812)
Borrmann type	5.425	0.02	1.154 (1.023,1.302)

**Table 4:** Multivariate Cox stepwise proportional hazard test for overall survival in 1800 gastric cancer patients with gastrectomy.

Variable	?2	P value	Hazard ratio (95% CI)
Age	13.402	<0·001	1.296 (1.128, 1.489)
Tumor size	48.561	<0·001	1.724 (1.479,2.010)
Lymph node metastasis	160.489	<0·001	1.448 (1.368.1.534)
Depth of invasion	42.172	<0·001	1.306 (1.205,1.416)
Curability	50.753	<0·001	1.718 (1.480,1.994)

### Result 3: Stage-stratified Analysis of Prognostic Factors, According to Tumor Size

**Figure 4:**
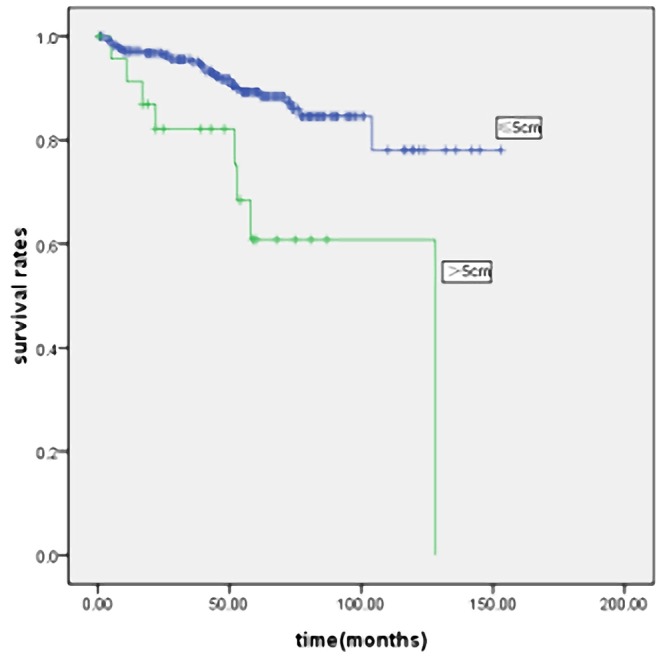
Survival curves for the 318 patients with stage I gastric cancer according to the tumor size (p = 0.000).

**Figure 5:**
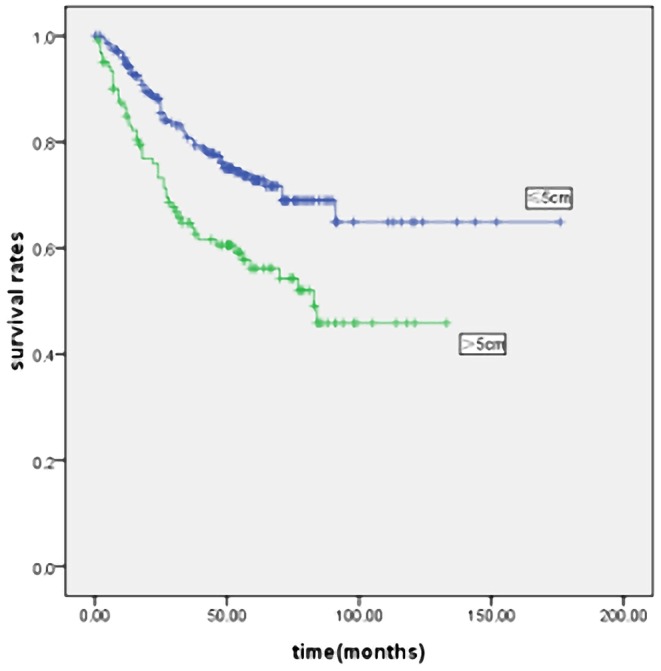
Survival curves for the 387 patients with stage II gastric cancer according to the tumor size (p = 0.000).

**Figure 6:**
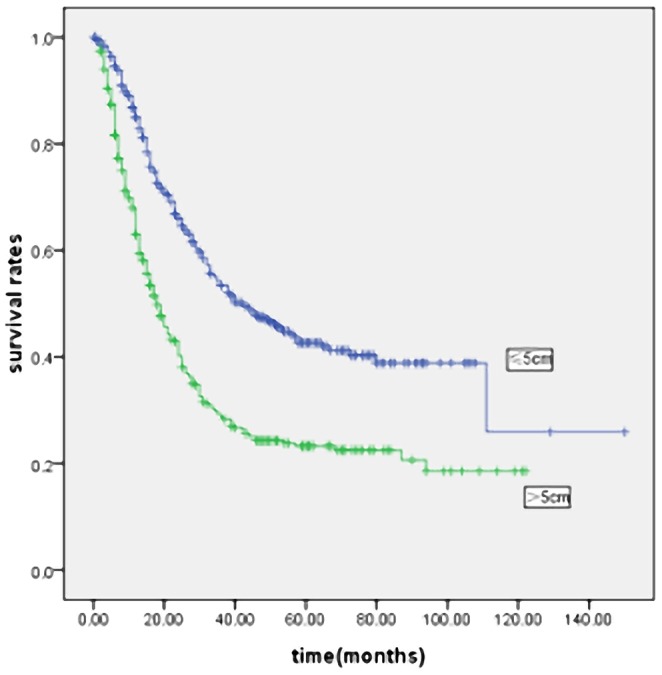
Survival curves for the 831 patients with stage III gastric cancer according to the tumor size (p = 0.000).

**Figure 7:**
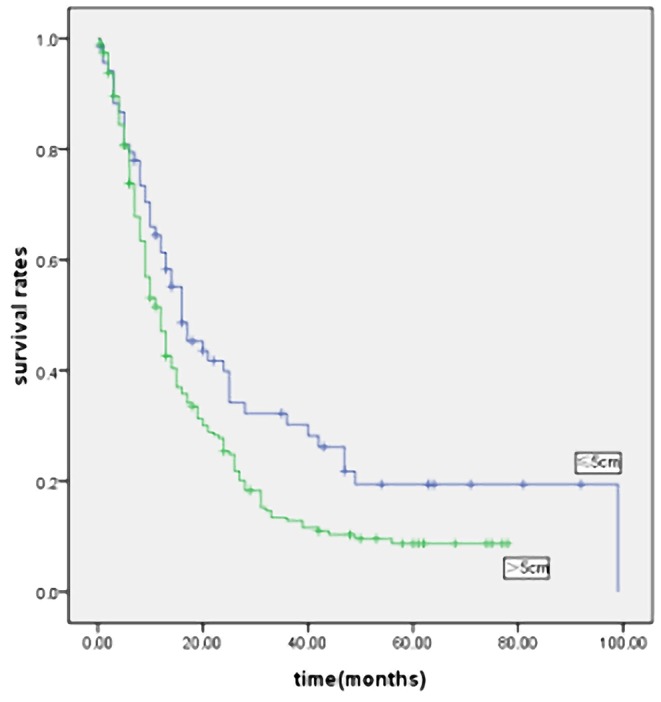
Survival curves for the 264 patients with stage IV gastric cancer according to the tumor size (p = 0.014).

In order to eliminate the influence of stage on prognosis, we performed a stage-stratified analysis of all patients according to tumor size. Of the 1800 patients, 318 patients (17.6%) had stage I disease, 387 patients (21.5%) had stage II disease, 831 patients (46.2%) had stage III disease, and 264 patients (14.7%) had stage IV disease. We found that tumor size significantly affected the survival in patients with stage I, II, III and IV disease (Figures 4, 5, 6 and 7). Five-year survival rates for each stage in patients with SSG were significantly better than in patients with LSG. Tumor size also affected survival in patients with stages T2, T3 and T4 and stages N0, N1, N2 and N3. Patients with LSG had a significantly poorer survival than those with SSG and the same depth of tumor invasion (except for patients with stage T1 disease). The prognosis of LSG was worse than that of SSG, in patients with a similar number of lymph node metastases. In order to balance the impact of differences in degree of differentiation, Borrmann type, location, stage, age or curability, we carried out a pT, pN and pTNM stage-stratified analysis according to tumor size, using a multivariate cox stepwise proportional hazards test. Tumor size was an independent prognostic factor in patients with stages T2, T3, T4; stages N0, N1, N2 and N3; and stages I, II, III and IV (Tables S1, S2 and S3).

## Discussion

We adopted a 5 cm cut-off value for tumor size and found that patients with LSG had more aggressive features than patients with SSG. Tumor size was an independent prognostic indicator in patients with gastric cancer, regardless of type of surgery. Significant differences in survival were identified between SSG and LSG patients in stages I, II, III and IV. Patients with LSG gastric cancer had more disease progression and a worse prognosis. Tumor size was not only was an indicator of tumor grade or local invasion, but also was a prognostic indicator of patient survival.

Tumor diameter is easily measured during surgery. Tumor size has been used as a staging criterion in breast, thyroid and lung cancer. The UICC (international Union against Cancer) and GRGCS (General Rules for Gastric Cancer Study of Japan) have not included tumor size as a prognostic factor in staging gastric cancer. The prognostic value of tumor size in gastric cancer has been reported previously. A cutoff of tumor size has been used by different researchers. Saito et al [11] reported a tumor size of 8 cm gave the best breakpoint for predicting prognosis, and a multivariate analysis showed that tumor size was an independent prognostic factor. Adachi et al [7] divided patients into three groups: <4 cm, 4–10 cm, and >10 cm in diameter. Survival rates decreased with increasing tumor size. In a series of 273 pT3 gastric cancer patients, Xiaowen Liu and Yu Xu et al [12] reported that tumor size was an independent prognostic factor when classified at a cutoff value of 6 cm. After univariate and multivariate analysis, we adopted a 5 cm cut-off value for tumor size and found that tumor size was significantly related to the prognosis of gastric carcinoma. Univariate analysis showed that the prognosis of patients with tumor size ≤5 cm was significantly better than that of patients with tumor size >5 cm. These results agree with previous studies [8], [9], [13]. The poor outcomes associated with LSG may be attributed their aggressive features and more advanced stages. LSG patients were characterized by more frequent location in the entire of the stomach (18.5% vs. 1.9%), large proportion of Borrmann type IV (12.4% vs. 4.0%), and more mucinous carcinoma cell type (9.4% vs. 4.7%). As we previously reported [14], the overall 5-year survival of patients with mucinous gastric cancer was 27.2%, compared to 42.8% in patients with no mucinous component in their gastric cancer. Kitamura K and Maehara Y et al [15], [16] found that Borrmann type IV gastric cancer patients were diagnosed at an advanced stage, and had a very poor prognosis. We found that LSG were more prone to metastasize to the liver than SSG. This may be due to the ability of LSG to more easily by spread by lymphatics or direct invasion. Our study showed that larger tumors were significantly associated with deeper depth of invasion, and higher incidence of lymph node metastases (P<0.0001), consistent with other reports [17], [18], [19]. The aggressive features of LSG (higher grade and increased rate of local invasion) results in a larger percentage of patients with stage IV disease (25.8% vs. 6.6%), and a higher proportion of total gastrectomy and combined organ resections performed.

Depth of invasion is an important prognostic predictor in patients with gastric cancer. To address the confounding influence of T stage, we performed a T-stratified analysis according to tumor size. For tumors limited to the same depth of invasion (T2, T3 and T4), the prognosis of LSG patients was significantly worse than that of SSG patients. Multivariate analysis showed that the presence of lymph node metastases was the only independent prognostic factor affecting survival in patients with stage T1 (Table S1). Patients we saw with stage T1 did not have an independent relation between tumor size and survival, similar to Kikuchi et al [20]. In their report, lymph node metastases were an independent prognostic indicator. It is likely that lymph node metastases are more important than tumor size in predicting prognosis in early gastric cancer. Lymph node resection is important in these patients. Chen Li et al [21] reported that the prognosis of patients with large tumors was significantly worse than that of patients with small tumors in advanced gastric cancer. We had similar findings. The survival of LSG was worse than that of SSG in patients with advanced gastric cancer. In patients with early gastric cancer, the survival of patients was less influenced by tumor size. Tumor size should be included in T stage to better predict patient prognosis.

Lymph node status is an important prognostic indicator in patients with gastric cancer. In our N stage analysis, stratified according to tumor size, tumor size was significantly related to patient survival in those with stage N0, N1, N2 and N3. Five-year survival rates in patients with LSG were significantly worse than in patients with SSG. Multivariate analysis revealed that tumor size was an independent prognostic factor in patients with stage N0, N1, N2 and N3 (Table S2). Tumor size affected survival in both node-negative and node-positive patients. Dong Yi Kim et al [22] found that tumor size was the only independent, significant factor for the prediction of long-term survival in node-positive gastric carcinoma patients after curative resection. The size of the tumor has also been reported to be a significant prognostic factor for survival in node-negative gastric cancer patients [23], [24], [25]. This was confirmed in our patients. Youichi Kikuchi et al [26] reported that tumor size was related to the incidence of lymph node micrometastases in clinically node negative patients. Lymph nodes dissection in patients with tumors larger than 5 cm may improve their survival.

Patient outcome after curative surgery was significantly better than after non-curative surgery. LSG patients we operated on had a higher proportion of non-curative surgery (39.3% vs. 17.9%). The decreased relative survival of LSG might be attributed to the higher percent of non-curative patients. We performed curability-stratified and stage-stratified analyses to better evaluate this question. The survival of LSG patients was worse than that of SSG patients after curative surgery. In the TNM stage-stratified analysis of prognostic factors, stratified according to tumor size, we found that tumor size was significantly associated with survival in patients with stages I, II, III and IV. Five-year survival rates for these stages in patients with SSG were significantly better than in patients with LSG (Figures 4, 5, 6 and 7). This confirmed that the prognosis of patients after curative surgery (stages I, II and III) and non-curative surgery (stage IV) was similarly influenced by the tumor size. Multivariate analysis revealed that tumor size was an independent prognostic factor in patients with stages I, II, III and IV disease (Table S3).

It could be questioned whether the measurements of fixed specimens represents the actual size of the tumors. Shrinkage occurs during fixation, and is more evident in the surrounding normal tissue than in the tumor itself. For the most part, the maximum diameter of tumors measured after fixation accurately represents an intraoperative evaluation [8].

One limitation of this study was the lack of data regarding adjuvant chemotherapy. Because of this deficiency, we did not evaluate the potential survival benefit that might be related to adjuvant chemotherapy. Adjuvant chemotherapy could potentially reduce lymph node micrometastases and improve disease-free survival. Toru Aoyama et al [27] reported that tumor diameter was the most important prognostic factor for survival in patients with stage II/III gastric cancer who underwent D2 gastrectomy followed by adjuvant S-1 chemotherapy. A prospective clinical study should be performed to assess the survival benefit of chemo-therapy in patients with larger tumor size and advanced gastric cancer.

In conclusion, when we adopted a tumor size cutoff of 5 cm, we had the following findings: (1). LSG patients had more aggressive clinico-pathologic features than SSG patients and tumor size was an independent prognostic indicator for patients with gastric cancer; (2). tumor size was significantly associated with the depth of invasion and presence of lymph node metastases. PT and pN stage-stratified analysis showed the survival of LSG was worse than that of the SSG in patients with advanced gastric cancer. In patients with early gastric cancer, the survival of patients was less influenced by tumor size. The prognosis of patients with LSG was worse than that of SSG, in patients with a similar number of lymph node metastases. (3). In the TNM stage-stratified analysis of prognostic factors, tumor size significantly affected survival in patients with stages I, II, III and IV. Tumor size may be of value in evaluating the prognosis of gastric carcinoma. Tumor size may help supplement clinical staging and guide improvements in the treatment of patients with gastric carcinoma.

## Supporting Information

Table S1 Multivariate cox stepwise proportional hazard test for overall survival in patients by T stage.
(DOC)

Table S2 Multivariate cox stepwise proportional hazard test for overall survival in patients by N stage.
(DOC)

Table S3 Multivariate cox stepwise proportional hazard test for overall survival in patients by TNM stage.
(DOC)
