# Correction: Stage-Stratified Analysis of Prognostic Significance of Tumor Size in Patients with Gastric Cancer

**DOI:** 10.1371/journal.pone.0109497

**Published:** 2014-09-26

**Authors:** The PLOS ONE Staff

The affiliation for the first author is incorrect. Hongliang Zu is also affiliated with: Department of General Surgery, Daqing Oilfield General Hospital, Daqing, Heilongjiang Province, China.
