# Supplementary material for: Stage-Stratified Analysis of Prognostic Significance of Tumor Size in Patients with Gastric Cancer
Source: PLoS One. 2013 Jan 30;8(1):e54502. doi: 10.1371/journal.pone.0054502 (PMC3559879; doi:10.1371/journal.pone.0054502)
Supplement: Table S1 — Multivariate cox stepwise proportional hazard test for overall survival in patients by T stage. (DOC) [file pone.0054502.s001.doc]

**Table S1 Multivariate cox stepwise proportional hazard test for overall survival in patients by T stage.**

| **Stage T** | **Variable** | **χ2** | **P value** | **Hazard ratio (95% CI)** |
| --- | --- | --- | --- | --- |
| *T1（n=138）* | *Lymph node metastasis* | *4.963* | *0.026* | *1.948（1.083,3.501）* |
| *T2（n=408）* | *Tumor size* | *21.788* | *﹤0·001* | *2.524（1.711,3.724）* |
|  | *Lymph node metastasis* | *28.007* | *﹤0·001* | *1.592（1.340,1.892）* |
|  | *Curability* | *19.074* | *﹤0·001* | *2.488 (1.653,3.745)* |
|  | *Age* | *15.189* | *﹤0·001* | *2.141(1.460,3.139)* |
| *T3（n=922）* | *Tumor size* | *33.084* | *﹤0·001* | *1.737(1.439,2.097)* |
|  | *Lymph node metastasis* | *108.307* | *﹤0·001* | *1.499(1.389,1. 618)* |
|  | *Curability* | *18.622* | *﹤0·001* | *1.569(1.279,1.925)* |
|  | *Age* | *8.154* | *0.004* | *1.307（1.088,1.570）* |
| *T4（n=332）* | *Tumor size* | *4.185* | *0.041* | *1.406(1.014,1.949)* |
|  | *Lymph node metastasis* | *23.647* | *﹤0·001* | *1.297(1.168,1.441)* |
|  | *Curability* | *13.928* | *﹤0·001* | *1.681(1.280,2.208)* |
