# Supplementary material for: Stage-Stratified Analysis of Prognostic Significance of Tumor Size in Patients with Gastric Cancer
Source: PLoS One. 2013 Jan 30;8(1):e54502. doi: 10.1371/journal.pone.0054502 (PMC3559879; doi:10.1371/journal.pone.0054502)
Supplement: Table S2 — Multivariate cox stepwise proportional hazard test for overall survival in patients by N stage. (DOC) [file pone.0054502.s002.doc]

**Table S2 Multivariate cox stepwise proportional hazard test for overall survival in patients by N stage.**

| **Stage N** | **Variable** | **χ2** | **P value** | **Hazard ratio (95% CI)** |
| --- | --- | --- | --- | --- |
| *N0（n=609）* | *Tumor size* | *17.951* | *﹤0·001* | *2.185(1.522,3.137)* |
|  | *Depth of invasion* | *14.341* | *﹤0·001* | *1.580(1.247,2.002)* |
|  | *Age* | *4.554* | *0.033* | *1.446(1.031,2.028)* |
| *N1（n=385）* | *Tumor size* | *5.778* | *0.016* | *1.513(1.079,2.120)* |
|  | *Depth of invasion* | *18.586* | *﹤0·001* | *1.735(1.350,2.229)* |
|  | *Curability* | *19.766* | *﹤0·001* | *2.192(1.551,3.098)* |
|  | *Age* | *13.203* | *﹤0·001* | *1.821(1.318,2.156)* |
| *N2（n=408）* | *Tumor size* | *15.846* | *﹤0·001* | *1.764(1.334,2.333)* |
|  | *Depth of invasion* | *8.994* | *0.003* | *1.392(1.121,1.728)* |
|  | *Curability* | *20.268* | *﹤0·001* | *1.888(1.432,2.491)* |
|  | *Age* | *6.395* | *0.011* | *1.393(1.077,1.800)* |
| *N3（n=398）* | *Tumor size* | *19.115* | *﹤0·001* | *1.773（1.372,2.292）* |
|  | *Curability* | *16.132* | *﹤0·001* | *1.637（1.287,2.081）* |
