# Supplementary material for: Stage-Stratified Analysis of Prognostic Significance of Tumor Size in Patients with Gastric Cancer
Source: PLoS One. 2013 Jan 30;8(1):e54502. doi: 10.1371/journal.pone.0054502 (PMC3559879; doi:10.1371/journal.pone.0054502)
Supplement: Table S3 — Multivariate cox stepwise proportional hazard test for overall survival in patients by TNM stage. (DOC) [file pone.0054502.s003.doc]

**Table S3. Multivariate cox stepwise proportional hazard test for overall survival in patients by TNM stage.**

| **Stage TNM** | **Variable** | **χ2** | **P value** | **Hazard ratio (95% CI)** |
| --- | --- | --- | --- | --- |
| *Stage Ⅰ(n=318)* | *Tumor size* | *4.634* | *0.031* | *2.659（1.092,6.478）* |
| *Stage Ⅱ(n=387)* | *Tumor size* | *15.758* | *﹤0·001* | *2.183（1.485,3.210）* |
|  | *Curability* | *5.903* | *0.015* | *1.791 (1.119,2.866)* |
| *Stage Ⅲ(n=831)* | *Tumor size* | *40.901* | *﹤0·001* | *1.839（1.526,2.216）* |
|  | *Curability* | *30.928* | *﹤0·001* | *1.741(1.432,2.117)* |
|  | *Borrmann type* | *6.581* | *0.01* | *1.195(1.043,1.368)* |
|  | *Age* | *8.408* | *0.004* | *1.311(1.092,1.575)* |
| *Stage Ⅳ(n=264)* | *Tumor size* | *3.898* | *0.048* | *1.352(1.002,1.823)* |
|  | *Curability* | *4.030* | *0.045* | *1.407（1.008,1.964）* |
